# Ultrasensitive detection of uveal melanoma using [^18^F]AlF-NOTA-PRGD2 PET imaging

**DOI:** 10.1186/s13550-024-01123-4

**Published:** 2024-07-05

**Authors:** Ling Wang, Xue Zhu, Yan Xue, Zhihong Huang, Wenjun Zou, Zhengwei Zhang, Mengxi Yu, Donghui Pan, Ke Wang

**Affiliations:** 1 NHC Key Laboratory of Nuclear Medicine, Jiangsu Key Laboratory of Molecular Nuclear Medicine, Jiangsu Institute of Nuclear Medicine, Wuxi, 214063 Jiangsu Province China; 2 Department of Radiopharmaceuticals, School of Pharmacy, Nanjing Medical University, Nanjing, 211166 Jiangsu Province China; 3 Department of Ophthalmology, Jiangnan University Medical Center JUMC, Wuxi No.2 People’s Hospital, Wuxi, 214000 Jiangsu Province China

**Keywords:** Uveal melanoma, **[**^18^F]AlF-NOTA-PRGD2, [^18^F]FDG, PET imaging

## Abstract

**Background:**

Uveal melanoma (UM) is the most common primary intraocular tumor in adults, and early detection is critical to improve the clinical outcome of this disease. In this study, the diagnostic effectiveness of [^18^F]AlF-NOTA-PRGD2 (an investigational medicinal product) positron emission tomography (PET) imaging in UM xenografts and UM patients were evaluated. The cell uptake, cell binding ability and in vitro stability of [^18^F]AlF-NOTA-PRGD2 were evaluated in 92-1 UM cell line. MicroPET imaging and biodistribution study of [^18^F]AlF-NOTA-PRGD2 were conducted in 92-1 UM xenografts. Then, UM patients were further recruited for evaluating the diagnostic effectiveness of [^18^F]AlF-NOTA-PRGD2 PET imaging (approval no. NCT02441972 in clinicaltrials.gov). In addition, comparison of [^18^F]AlF-NOTA-PRGD2 and ^18^F-labelled fluorodeoxyglucose ([^18^F]FDG) PET imaging in UM xenografts and UM patients were conducted.

**Results:**

The in vitro data showed that [^18^F]AlF-NOTA-PRGD2 had a high cell uptake, cell binding ability and in vitro stability in 92-1 UM cell line. The in vivo data indicated that 92-1 UM tumors were clearly visualized with the [^18^F]AlF-NOTA-PRGD2 tracer in the subcutaneous and ocular primary UM xenografts model at 60 min post-injection. And the tumor uptake of the tracer was 2.55 ± 0.44%ID/g and 1.73 ± 0.15%ID/g at these two tissue locations respectively, at 7 days after animal model construction. The clinical data showed that tumors in UM patients were clearly visualized with the [^18^F]AlF-NOTA-PRGD2 tracer at 60 min post-injection. In addition, [^18^F]AlF-NOTA-PRGD2 tracer showed higher sensitivity and specificity for PET imaging in UM xenografts and UM patients compared to [^18^F]FDG tracer.

**Conclusion:**

[^18^F]AlF-NOTA-PRGD2 PET imaging may be a more preferred approach in the diagnosis of primary UM compared to [^18^F]FDG PET imaging. Additionally, due to the high tumor-to-background ratio, [^18^F]AlF-NOTA-PRGD2 PET imaging seems also to be applicable for the diagnosis of UM patients with liver metastasis.

*Trial registration*: ClinicalTrials.gov: NCT02441972, Registered 1 January 2012, https://clinicaltrials.gov/study/NCT02441972.

## Introduction

Although rare, uveal melanoma (UM) is the primary intraocular tumor worldwide [1, 2]. Control of local UM seems effective through non-pharmacological treatment strategies, but up to 50% of patients still develop metastasis. Overall, with a very poor prognosis [3, 4], early detection and timely treatment are critical to improve the clinical outcome of UM [5]. Currently, UM diagnosis relies on clinical examination, the slit lamp and indirect ophthalmoscope, ocular ultrasonography, magnetic resonance imaging (MRI) and positron emission tomography/computerized tomography (PET/CT) of whole body [6, 7]. However, the primary challenge is to distinguish small ocular tumors with a thickness of less than 3 mm, from presumed naevi as well as to detect metastasis in the whole body. PET/CT as a traditional non-invasive technique, plays a critical role in oncologic imaging and has been widely used for the diagnosis and staging of a variety of malignancies [8–10]. The sensitivity and specificity of PET scanning largely relies on the imaging tracer. In the past 40 years, [^18^F]FDG is the dominant PET tracer used in the imaging of neurology, cardiology, and oncology [11–14]. However, the efficacy of [^18^F]FDG PET imaging in the detection of UM is very poor with a high incidence of false-negative results. The limited sensitivity of [^18^F]FDG PET imaging in detection of small size tumor is due to low metabolic nature of UM. In addition, it is also not sensitive enough for the detection of UM metastases into the liver and other tissues. In contrast, liver metastases from cutaneous melanoma are shown to be FDG sensitive [15, 16]. Therefore, to develop a more sensitive PET imaging tracer is helpful for a better differential diagnosis and staging of UM, which can be used as a supplement routine examination.

Integrins [consisting of two noncovalently bound transmembrane subunits (*α* and β)] are large, membrane-spanning, heterodimeric proteins [17, 18]. Integrin alphaVbeta3 (integrin αvβ3) is one member of integrins and contributes to biological processes such as cell adhesion and cell migration [19]. In the past decade, integrin αvβ3 was adopted as an important molecular target for early and differential diagnosis of rapidly growing solid tumors due to its role in tumor angiogenesis [20, 21]. Integrin αvβ3 is a receptor for arginine-glycine-aspartic (RGD) tripeptide, which also includes linear and cyclic RGD peptide antagonists. Thus, targeting integrin αvβ3 is a viable approach to develop radiotracers for PET/CT [22]. Notni et al. have conducted a preclinical evaluation of [^68^Ga]TRAP(RGD)_3_ in human melanoma xenografts and showed that there is a high uptake of such radiotracer [23]. Chen et al. develop a new PET imaging probe [^18^F]AlF-NOTA-PRGD2 (NOTA-PEG4-E[c(RGDfK)]2) and successfully applied to lung cancer patients, with longer tumor retention and simpler labeling process [24]. Up to date, [^18^F]AlF-NOTA-PRGD2 tracer has not been applied to PET imaging of UM patients yet.

The previous studies and our data have confirmed integrin αvβ3 is highly expressed in UM cells and UM tissues [25]. And the current study is the first to evaluate the efficacy of [^18^F]AlF-NOTA-PRGD2 for PET imaging of UM, in comparison to that of [^18^F]FDG.

## Materials and methods

### General materials

All commercially obtained chemicals were analytical grade and used without further purification. FDG (fluorodeoxyglucose) was provided from the Wuxi Jiangyuan Industrial Technology and Trade Corporation and reconstituted with sterile saline. PRGD2 (PEG4-E[c(RGDyK)]2) and NOTA-PRGD2 were provided from Chinese Peptide Company (Hangzhou, China). ^18^F-fluoride was obtained from an in-house PET trace cyclotron (HM-7, Sumitomo Heavy Industries Ltd, Japan) via the ^18^O (p, n)^18^F nuclear reaction.

### Radiosyntheses of [^18^F]FDG and [^18^F]AlF-NOTA-PRGD2

[^18^F]FDG and [^18^F]AlF-NOTA-PRGD2 were synthesized as the previous report [26]. The preparation of [^18^F]AlF-NOTA-PRGD2 was solved as follows: NOTA-PRGD2 (50 µg contains 1.6 µg AlCl_3_) add 20 µL water to dissolved in the reaction bottle and add 5 µL of glacial acetic acid to adjust PH (PH ≈ 3). Then, 20 μL [^18^F] fluorinated water (~ 1110 MBq) and 0.2 mL acetonitrile were added to the reaction flask. The above solution was reacted at 95 °C for 10 min, the product was diluted with 9 mL water and hung on an activated Varian BOND ELUT C18 column. Wash the C18 column three times with 10 mL water. Finally, 300 µL of 10 mM hydrochloric acid was used to elute the product in ethanol. The mixture was purified by the C18 cartridge using High Performance Liquid Chromatography (HPLC). The final product was formulated in saline for the subsequent studies.

### Cell culture and UM xenografts

Human UM cell lines (92-1, OCM-1A and MEL270) were obtained from OcuTech Co., Ltd (Wuxi, China) and cultured in RPMI1640 medium with 10% FBS (fetal bovine serum) and 1% P/S (penicillin/streptomycin). The expression of integrin αvβ3 on 92-1 cells were confirmed by western blot analysis and immunofluorescence. All experiment protocols were approved by the Animal Care and Use Committee of Jiangsu Institute of Nuclear Medicine (JSINM-2022-061). The male BALB/c nude mice (4–5 weeks old, 18–22 g body weight) were purchased from Cavens Laboratory Animal Technology Co. Ltd. (Jiangsu, China). The nude mice were injected with 5 × 10^6^ 92-1-Luc cells in subcutaneous or 1 × 10^5^ 92-1-Luc cells in ocular, when the tumor size reaches about 200 mm^3^, about 3–4 weeks after the cell inoculation, the tumor model could be used for further study.

### Western blot analysis

Cells were lysed with RIPA (Radio Immunoprecipitation Assay) buffer and protein concentration was determined with BCA assay (Beyotime, Nantong, China). Protein extract (20 μg) was separated by SDS-PAGE and electrophoretically transferred to polyvinylidene fluoride (PVDF) membrane. The membrane was incubated with the primary antibody of integrin alpha V (ab179475, Abcam, MA, USA) and integrin beta 3 (ab179473, Abcam, MA, USA) followed by the horseradish peroxidase-conjugated secondary antibody (ab97051, Abcam, MA, USA). The expression of target protein was verified by chemiluminescence (ECL) detection kit. Band densities was analyzed by Image J (NIH, MD, USA) with normalization to that of GAPDH.

### Immunofluorescence analysis

Cells were fixed by 4% paraformaldehyde (15 min) and closed by QuickBlock™ Blocking Buffer for Immunol Staining (1 h) (P0260, Beyotime, Nantong, China) at room temperature. Then, cells were incubated by integrin αvβ3 antibody (abs122318, Absin, China) at 4 °C for 24 h, followed by Alexa Fluor 488-conjugated goat anti-rabbit IgG (1 h) at room temperature. DAPI was used for nuclei staining. Fluorescence was observed by microscope (Olympus IX53, Olympus Corporation, Tokyo, Japan).

### Hematoxylin–eosin staining

The tumor and ocular tissues were dehydrated by using increased ethanol concentrations. Next, the tissue paraffin blocks were embedded. For the experiments, the paraffin sections (4 μm) were stained using hematoxylin for 5 min, and eosin for another 2 min.

### Cell uptake and binding assays

For cell uptake assay, cells (12-well plates, 1 × 10^5^ cells/well) were cultured for 24 h and then the culture medium were replaced by [^18^F]AlF-NOTA-PRGD2 (74 kBq/mL) containing medium (1 mL). The cells were incubated for 0.25 h, 0.5 h, 1 h, 2 h, washed with PBS three times and lysed by 0.1 M NaOH (1 mL). The radioactivity in the cells was measured by a γ-counter (Perkin-Elmer, MA, USA) and cell uptake was calculated. For cell binding assay, cells (24-well plates, 1 × 10^5^ cells/well) were cultured for 24 h, then the culture medium were replaced 2 mL medium of [^18^F]AlF-NOTA-PRGD2 (74 kBq/well) and unlabeled PRGD2 (the concentration ranges from 10^−2^ mM to 10^−10^ nM). After incubation for 2 h, cells were washed with PBS three times and lysed by NaOH (0.1 M, 1 mL) solution, and a γ-counter (Perkin-Elmer, MA, USA) was used for measuring the radioactivity. The value of IC50 was calculated by nonlinear regression analysis.

### In vitro stability test

The stability of [^18^F]AlF-NOTA-PRGD2 in different media was tested. [^18^F]AlF-NOTA-PRGD2 (37 MBq) was added to PBS or FBS (500 μL) solution, and incubated for 0, 1, 2 and 4 h at 37 °C, respectively. At the preselected time points, PBS samples and FBS samples (10 µL of 370 KBq) were directly analyzed with HPLC to measure radioactivity.

### MicroPET imaging and analysis

MicroPET scans and image analysis were performed using an Inveon microPET scanner (Siemens Medical Solutions, Germany). Mice were injected intravenously with 200 µL 3.7 MBq [^18^F]FDG or [^18^F]AlF-NOTA-PRGD2 (n = 4 per group). Under isoflurane anesthesia, 10-min static PET images were acquired at 1 h post-injection. The radioactivity value within the tumors, eye, brain, lung, heart, liver, spleen and kidney were obtained and ROI (regions of interest) was analyzed (%ID/g, percentage of injected dose per gram of tissues) using vendor software (ASI Pro 5.2.4.0). The value of tumor-to-organ was also calculated.

### Biodistribution study and analysis

The tumor-bearing mice (n = 4 per group) injected with [^18^F]FDG or [^18^F]AlF-NOTA-PRGD2 (3.7 MBq, 200 µL) were immediately sacrificed after microPET scan, tumors and major organs of tumor-bearing mice were collected and wet-weighed. A γ-counter (PerkinElmer, MA, USA) was used for measuring the radioactivity. The data were calculated and expressed as %ID/g.

### Clinical patients and PET imaging

This clinical study was approved by the ethics committee of The Hospital Affiliated to Jiangnan University (LS2011051). Four patients were enrolled in the clinical trial of [^18^F]AlF-NOTA-PRGD2 for cancer diagnostics (approval no.NCT02441972 in clinicaltrials.gov). All patients signed a written informed consent form. Two of the patients were clinically diagnosed as benign tumor by MRI. The other two patients were clinically diagnosed as suspected uveal melanoma by MRI. After PET/CT scan, two UM patients underwent eye enucleation within 7 days. Immunohistochemical staining data demonstrated that S100, melanoma gp100 (HMB45), MelanA were positive.

### Statistical analysis

Data are expressed as means ± SD. The data among different groups were compared using Student’s *t* test and one-way analysis of variance (ANOVA). As *p* < 0.05, statistically significant was considered.

## Results

### High expression of integrin αvβ3 in UM cells and UM xenografts

**Fig. 1:**
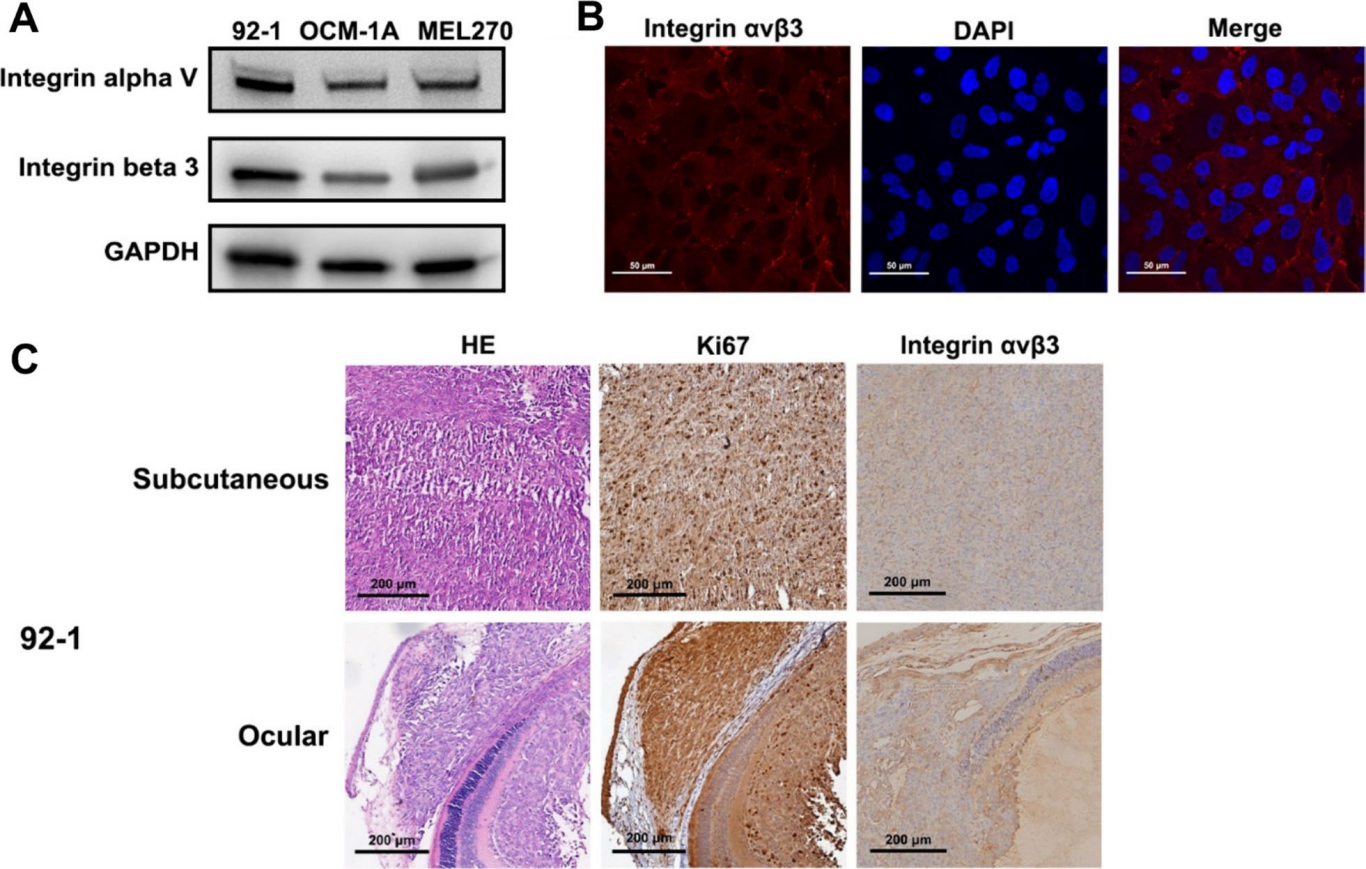
The expressions of integrin αvβ3 in UM cells and UM xenografts. **A** Western blot analysis of integrin αvβ3 in UM cell lines (92-1, OCM-1A and MEL270). **B** Immunofluorescent staining of integrin αvβ3 in 92-1 UM cells (integrin αvβ3: red fluorescence). **C** HE, Ki67 and IHC analysis of integrin αvβ3 in subcutaneous and ocular tumor tissues of 92-1 UM xenograft mice

The expressions of integrin αvβ3 in UM cell lines (92-1, OCM-1A and MEL270) were assessed with western blot analysis (Fig. 1A), and then its cellular localization in 92-1 cells was evaluated with immunofluorescent staining (Fig. 1B). The results showed that integrin αvβ3 was highly expressed in UM cell lines and mainly located at cell membrane in 92-1 UM cells. Moreover, high expressions of integrin αvβ3 were also observed in subcutaneous and ocular tumor tissues of the UM xenograft mice (Fig. 1C).

### Cell uptake, cell binding assays and in vitro stability test of [^18^F]AlF-NOTA-PRGD2

**Fig. 2:**
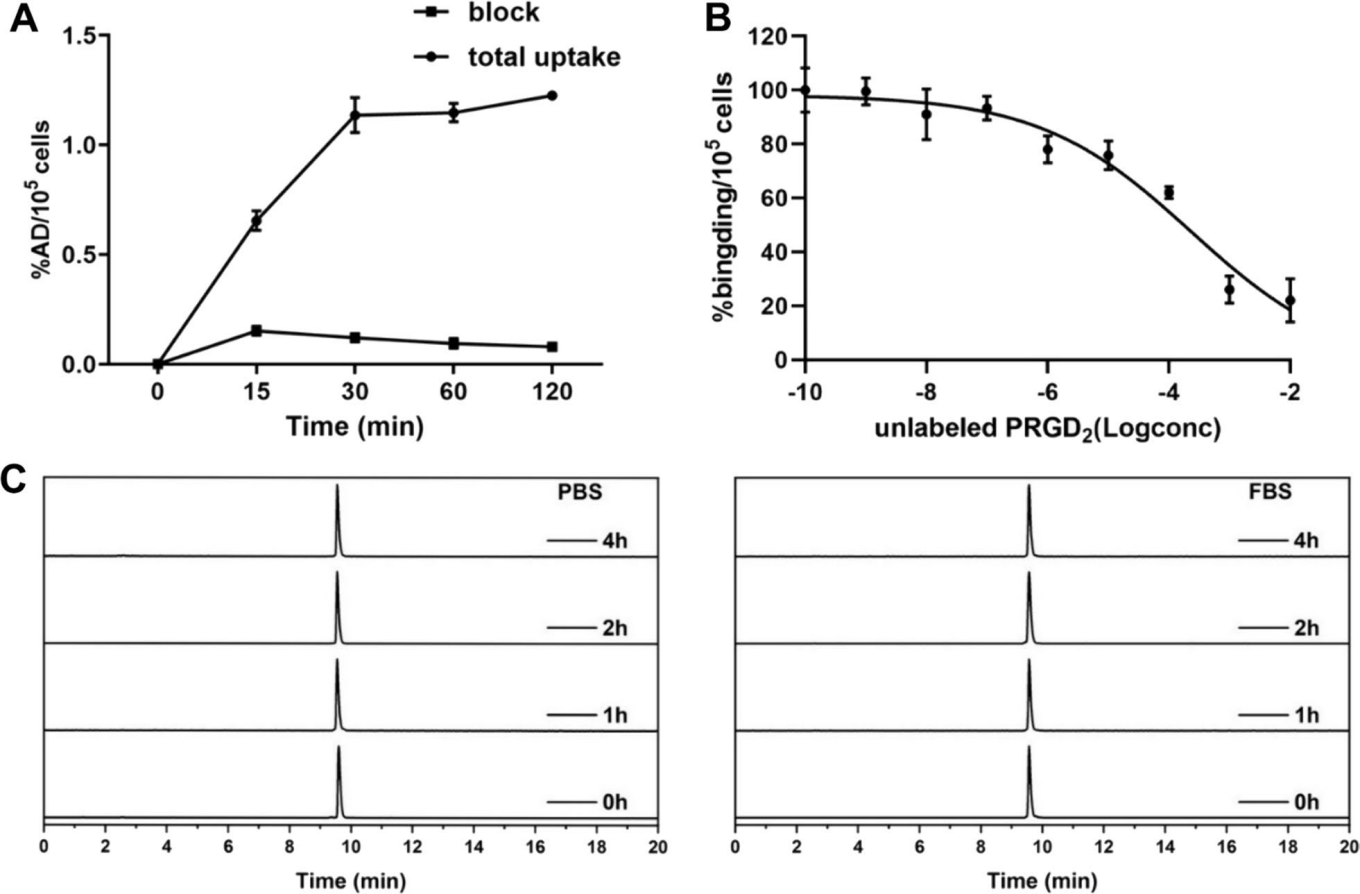
Cell uptake, cell binding assays and in vitro stability test of [^18^F]AlF-NOTA-PRGD2. **A** Cell uptake and block assays of [^18^F]AlF-NOTA-PRGD2 in 92-1 UM cells. **B** Competitive binding of [^18^F]AlF-NOTA-PRGD2 with unlabeled PRGD2. **C** In vitro stability assessment of [^18^F]AlF-NOTA-PRGD2 in PBS or FBS at different time points

The cell uptake of [^18^F]AlF-NOTA-PRGD2 was examined in 92-1 UM cells, which was gradually increased from 0.65 ± 0.04%AD (15 min after incubation) to 1.22 ± 0.08%AD (120 min after incubation) (Fig. 2A). Moreover, the uptake was effectively blocked in the presence of non-radiolabeled PRGD2. In cell binding assay, the unlabeled PRGD2 inhibited the binding of [^18^F]AlF-NOTA-PRGD2 to 92-1 UM cells in a dose-dependent manner with an IC_50_ value of 231.4 ± 1.95 nM (Fig. 2B). The in vitro stability of [^18^F]AlF-NOTA-PRGD2 was evaluated in PBS or FBS at 37 °C. [^18^F]AlF-NOTA-PRGD2 exhibited a good stability in both PBS or FBS for up to 4 h post-labelling, and no statistically significant change in radiochemical purity was observed throughout the duration (Fig. 2C). All the radiochemical purity were > 99%.

### [^18^F]AlF-NOTA-PRGD2 microPET imaging in UM xenografts and biodistribution study

**Fig. 3:**
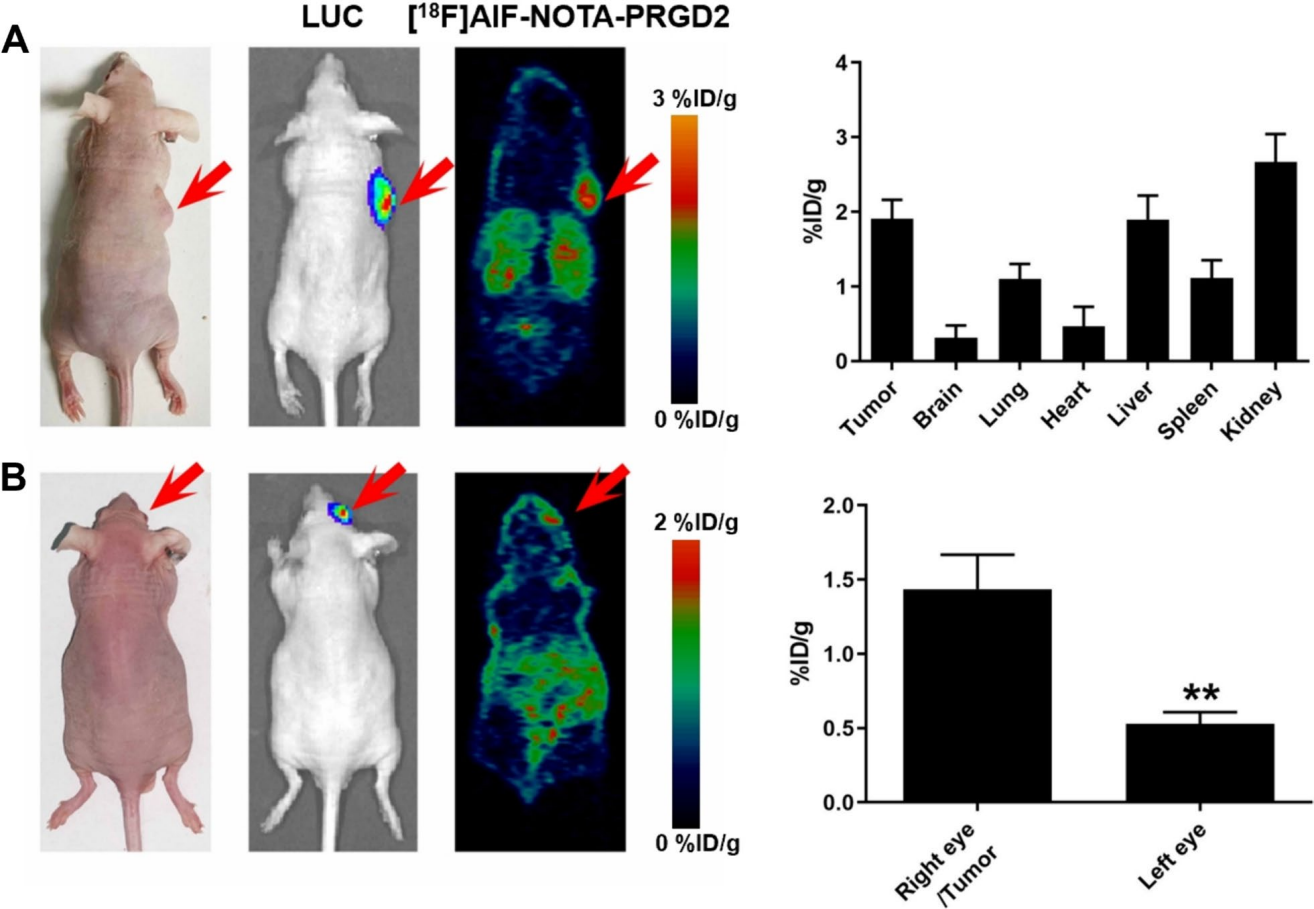
[^18^F]AlF-NOTA-PRGD2 microPET imaging in UM xenografts and biodistribution study. **A** In vivo microPET imaging of subcutaneous tumor in 92-1 UM xenografts with [^18^F]AlF-NOTA-PRGD2, and biodistribution analysis in whole body. **B** In vivo microPET imaging of ocular tumor in 92-1 UM xenografts with [^18^F]AlF-NOTA-PRGD2 and biodistribution analysis in eye. **p < 0.01 versus right eye/tumor

MicroPET study was performed using subcutaneous and ocular UM xenografts injected with 92-1-Luc cells, and 92-1-Luc tumor-bearing mice was used as a positive control. The subcutaneous and ocular UM xenografts were confirmed at Day 7 post injection by fluorescent imaging. MicroPET imaging was performed and UM tumors were clearly visualized with the [^18^F]AlF-NOTA-PRGD2 tracer at 60 min post-injection in both subcutaneous and ocular UM xenografts (Fig. 3). The tumor uptake is 2.55 ± 0.44%ID/g and 1.73 ± 0.15%ID/g, respectively. The 92-1-Luc UM tumor was clearly visualized with a good tumor-to-background. And the ROI analysis showed the quantitative tumor uptake and the accumulation in the liver and kidney as to microPET images. To confirm the localization of [^18^F]AlF-NOTA-PRGD2 in the 92-1-Luc UM tumor xenografts, biodistribution study was performed following microPET imaging. The tracer accumulation in tumors was 1.91 ± 0.25%ID/g and 1.44 ± 0.23%ID/g at 60 min post-injection. Its uptake in brain, lung, heart, liver, spleen and kidney in subcutaneous UM xenografts were 0.31 ± 0.17, 1.10 ± 0.20, 0.47 ± 0.26, 1.90 ± 0.32, 1.11 ± 0.24, 2.67 ± 0.37%ID/g, and which in ocular UM xenografts were 0.28 ± 0.06, 1.17 ± 0.24, 0.39 ± 0.03, 1.17 ± 0.12, 1.01 ± 0.15, 2.34 ± 0.39%ID/g. The biodistribution data were consistent with the ROI analysis of microPET imaging.

### Comparison of [^18^F]AlF-NOTA-PRGD2 and [^18^F]FDG microPET imaging in UM xenografts

**Fig. 4:**
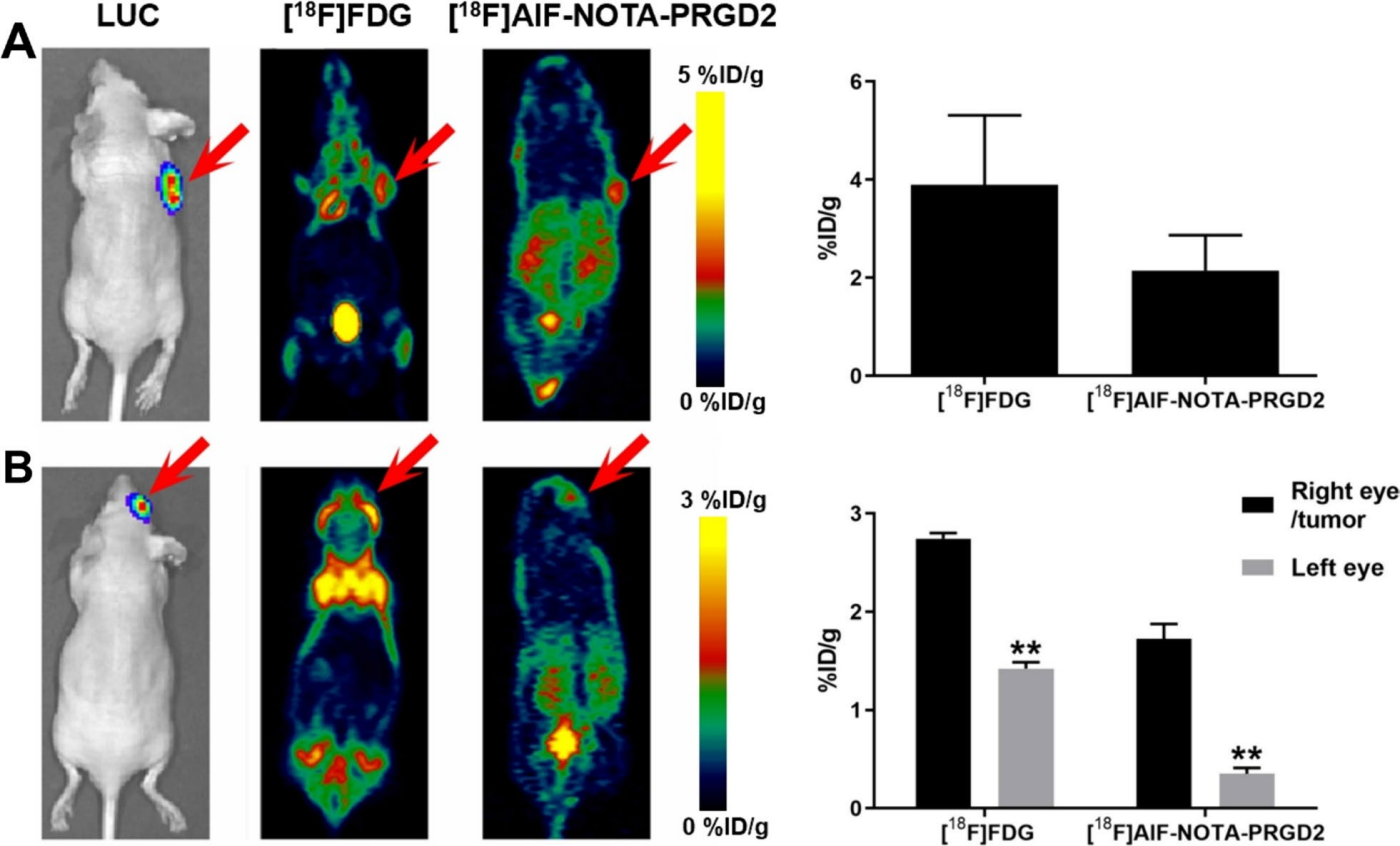
Comparison of [^18^F]AlF-NOTA-PRGD2 and [^18^F]FDG microPET imaging in UM xenografts. **A** In vivo microPET imaging of subcutaneous tumor in 92-1 UM xenografts using [^18^F]AlF-NOTA-PRGD2 and [^18^F]FDG in whole body. **B** In vivo microPET imaging of ocular tumor in 92-1 xenografts using [^18^F]AlF-NOTA-PRGD2 and [^18^F]FDG. **p < 0.01 versus right eye/tumor

The comparison of [^18^F]AlF-NOTA-PRGD2 and [^18^F]FDG microPET imaging in subcutaneous and ocular 92-1 UM xenografts was further conducted. Both of the two radiotracers were clearly visualized in subcutaneous tumors (Fig. 4). The signals of [^18^F]AlF-NOTA-PRGD2 tracer were distinctively observed in ocular 92-1 UM xenografts with very low background; while that of [^18^F]FDG was hard to be distinguished due to a strong brain uptake. The tumor-to-brain, -lung, -heart, -liver, -spleen and -kidney uptake ratios of [^18^F]AlF-NOTA-PRGD2 in subcutaneous UM xenografts were 6.45 ± 0.89, 1.71 ± 0.29, 4.18 ± 0.83, 1.01 ± 0.19, 1.74 ± 0.52 and 0.70 ± 0.11, respectively, while the corresponding ratios for [^18^F]FDG were 1.02 ± 0.33, 2.93 ± 0.65, 5.73 ± 1.84, 6.58 ± 1.97, 2.52 ± 0.54 and 6.32 ± 1.64, respectively (Table 1). The tumor-to-brain, -lung, -heart, -liver, -spleen and -kidney uptake ratios of [^18^F]AlF-NOTA-PRGD2 in ocular UM xenografts were 5.28 ± 0.49, 1.25 ± 0.28, 3.73 ± 0.48, 1.22 ± 0.12, 1.42 ± 0.06 and 0.61 ± 0.06, respectively, while the corresponding ratios for [^18^F]FDG were 0.89 ± 0.12, 2.09 ± 0.27, 2.71 ± 1.10, 4.06 ± 1.42, 2.18 ± 0.43 and 4.01 ± 0.68, respectively (Table 2).

**Table 1:** Subcutaneous tumor to organ ratios of [^18^F]AlF-NOTA-PRGD2 and [^18^F]FDG in UM xenografts

Organ	[^18^F]FDG	[^18^F]AlF-NOTA-PRGD2
Tumor to brain	1.02 ± 0.33	6.45 ± 0.89
Tumor to lung	2.93 ± 0.65	1.71 ± 0.29
Tumor to heart	5.73 ± 1.84	4.18 ± 0.83
Tumor to liver	6.58 ± 1.97	1.01 ± 0.19
Tumor to spleen	2.52 ± 0.54	1.74 ± 0.52
Tumor to kidney	6.32 ± 1.64	0.70 ± 0.11

**Table 2:** Ocular tumor to organ ratios of [^18^F]AlF-NOTA-PRGD2 and [^18^F]FDG in UM xenografts

Organ	[^18^F]FDG	[^18^F]AlF-NOTA-PRGD2
Tumor to left eye	1.72 ± 0.22	2.71 ± 0.05
Tumor to brain	0.89 ± 0.12	5.28 ± 0.49
Tumor to lung	2.09 ± 0.27	1.25 ± 0.28
Tumor to heart	2.71 ± 1.10	3.73 ± 0.48
Tumor to liver	4.06 ± 1.42	1.22 ± 0.12
Tumor to spleen	2.18 ± 0.43	1.42 ± 0.06
Tumor to kidney	4.01 ± 0.68	0.61 ± 0.06

### Comparison of [^18^F]AlF-NOTA-PRGD2 and [^18^F]FDG PET imaging in UM patients

**Fig. 5:**
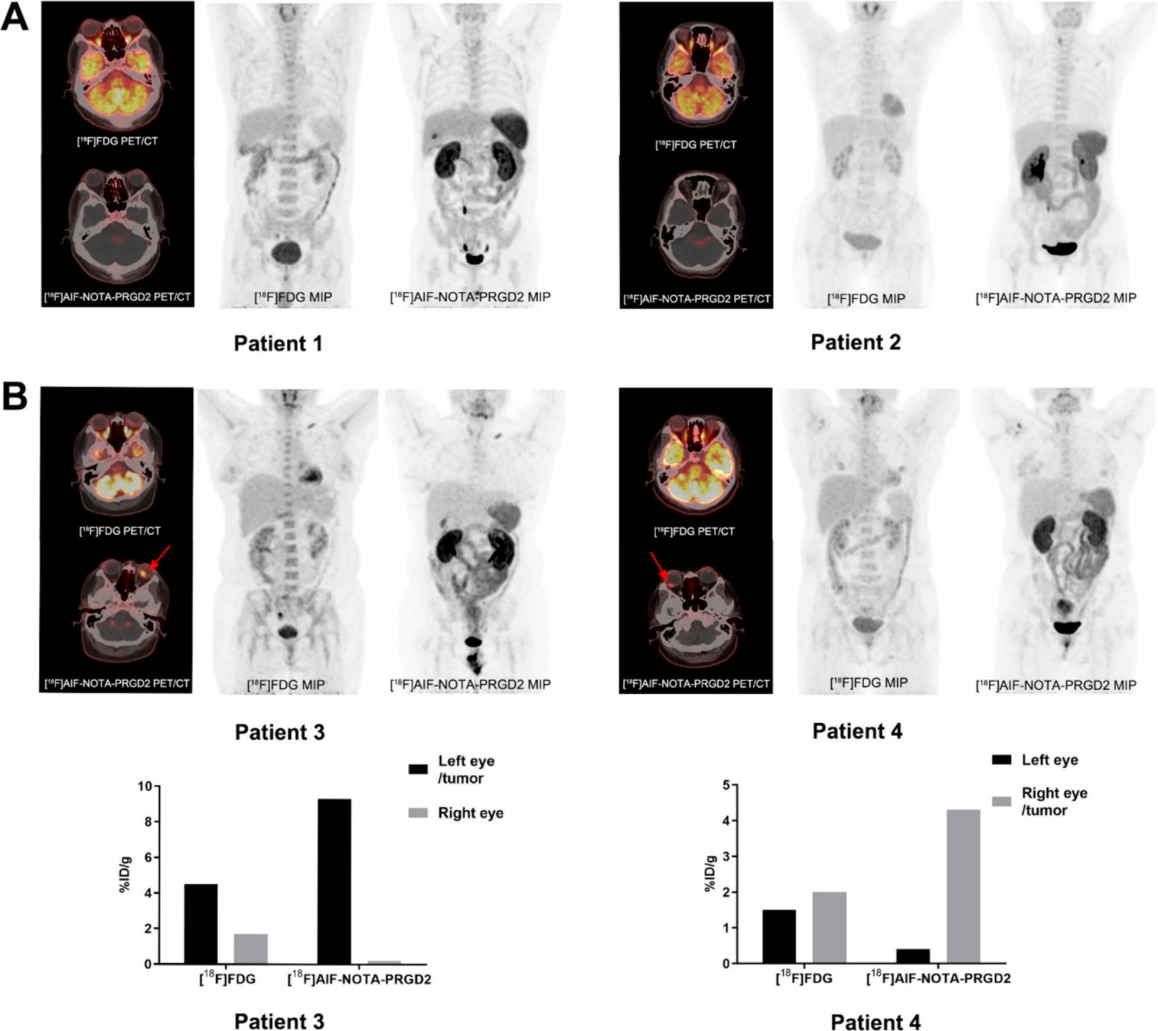
The PET imaging of [^18^F]AlF-NOTA-PRGD2 and [^18^F]FDG in patients. **A** Ocular PET imaging in Patient 1 and Patient 2 with benign tumor using [^18^F]AlF-NOTA-PRGD2 and [^18^F]FDG PET tracers. **B** Ocular PET imaging and tumor uptake in the Patient 3 and Patient 4 with UM using [^18^F]AlF-NOTA-PRGD2 and [^18^F]FDG PET tracers

The comparison of [^18^F]AlF-NOTA-PRGD2 and [^18^F]FDG PET imaging in two patients with primary UM was further determined. In the two patients with benign tumor, both two radiotracers had no detectable uptake (Fig. 5A). In the patients with UM, [^18^F]AlF-NOTA-PRGD2 demonstrated a lower background in the brain region than that of [^18^F]FDG, which was preferred for the detection of primary ocular UM tumor with a favorable target-to-background ratio (Fig. 5B). Such finding was consistent with the observation in UM xenografts. In addition, the tumor-to-brain, -lung, -heart, -liver, -spleen and -kidney uptake ratios of [^18^F]AlF-NOTA-PRGD2 were 46.50, 18.60, 7.15, 3.10, 1.33 and 0.14 for patient 3, and 14.33, 7.17, 2.87, 1.65, 0.67 and 0.44 for patient 4, respectively, while the corresponding ratios for [^18^F]FDG were 0.29, 6.43, 0.45, 1.41, 1.36 and 0.70 for patient 3, and 0.11, 2.86, 0.43, 0.56, 0.77 and 0.38 for patient 4, respectively (Table 3). The selectivity and sensitivity of [^18^F]AlF-NOTA-PRGD2 tracer for PET imaging of UM patients seems improved compared to that in UM xenografts.

**Table 3:** Ocular tumor to organ ratios of [^18^F]AlF-NOTA-PRGD2 and [^18^F]FDG in patient 3 and patient 4

Organ	Patient 3		Patient 4	
	[^18^F]FDG	[^18^F]AlF-NOTA-PRGD2	[^18^F]FDG	[^18^F]AlF-NOTA-PRGD2
Tumor to eye	2.65	46.50	1.33	10.75
Tumor to brain	0.29	46.50	0.11	14.33
Tumor to lung	6.43	18.60	2.86	7.17
Tumor to heart	0.45	7.15	0.43	2.87
Tumor to liver	1.41	3.10	0.56	1.65
Tumor to spleen	1.36	1.33	0.77	0.67
Tumor to kidney	0.70	0.14	0.38	0.44

## Discussion

In recent years, dimeric PRGD2 has been adopted as a new PET tracer in the early detection and staging of various tumors, due to its higher affinity for integrin αvβ3 receptor compared to monomeric counterpart [24, 27, 28]. In addition, compared with [^18^F]FDG, the radiosynthesis of this imaging tracer is convenient and less time consuming (< 30 min). The addition of a NOTA functional group to PRGD2 enabled a chelate compatible for both ^18^F-fluoride-aluminum and ^68^Ga. Li et al. report the effectiveness of [^68^Ga]PRGD2 PET/CT in glioma grading and demarcation [29]. Zheng et al. investigate the application of [^68^Ga]NOTA-PRGD2 PET/CT in lung cancer diagnosis, which showed a superior performance than that of [^18^F]FDG PET/CT due to specifically determining metastatic lymph nodes [30]. Zhang et al. show that [^18^F]AlF-NOTA-PRGD2 PET/CT noninvasively visualized GBM lesions and predicted the outcome of concurrent chemoradiotherapy as early as 3 weeks after the initiation of treatment [24]. However, there has been no report about the application of [^18^F]AlF-NOTA-PRGD2 in UM. This study first evaluated [^18^F]AlF-NOTA-PRGD2 PET imaging in the UM xenografts as well as a UM patients. Our data revealed that tumors in subcutaneous and ocular UM xenografts could be clearly visualized with this tracer at 60 min post-injection. Moreover, the PET imaging with this tracer in the UM patient may be more specific and sensitive than that in UM xenografts.

The characteristics of [^18^F]AlF-NOTA-PRGD2 were evaluated in in vitro and in vivo models of UM. Our in vitro studies revealed that [^18^F]AlF-NOTA-PRGD2 showed a high specificity to integrin αvβ3 in 92-1 UM cells in the aspects of cell uptake, high affinity in cell binding and high stability in vitro stability. The in vivo microPET imaging and biodistribution analysis demonstrated that [^18^F]AlF-NOTA-PRGD2 had a high tumor uptake and a very low background. In addition, integrin αvβ3 expression is upregulated during tumor angiogenesis, particularly in vascular endothelial cells. The high tumor uptake of [^18^F]AlF-NOTA-PRGD2 in vivo might also indicate the occurrence of tumor angiogenesis in UM. And the significantly increased liver and kidney uptake of [^18^F]AlF-NOTA-PRGD2 may suggest that this tracer is mainly metabolized in these two organs. As to PET imaging, [^18^F]AlF-NOTA-PRGD2 showed a longer retention in tumors and a faster clearance from normal tissues [31]. This study focuses on evaluating the application of [^18^F]AlF-NOTA-PRGD2 in the diagnosis of primary UM tumors; its application in metastatic UM xenografts needs to be assessed in the future studies.

[^18^F]FDG is the common PET tracer currently adopted in the early detection and staging of UM patients in clinical settings. However, a large variation in its sensitivity, specificity, and accuracy in UM diagnosis has been reported [16, 32]. Comparison of [^18^F]AlF-NOTA-PRGD2 and [^18^F]FDG in PET imaging showed the uptake of [^18^F]AlF-NOTA-PRGD2 in UM xenografts was lower than that of [^18^F]FDG; but this was opposite in the UM patients. Moreover, the low background of [^18^F]AlF-NOTA-PRGD2 in the brain region promoted its application in the detection of primary UM tumor with a high target-to-background ratio. In addition, the tumor to organ ratios of [^18^F]AlF-NOTA-PRGD2 in the UM patients were superior than that in UM xenografts, in particular the ratio of tumor-to-live, which made this probe to be suitable for the diagnosis of UM patients with live metastasis. It is speculated that ^18^F]AlF-NOTA-PRGD2 is more easily metabolized in the human’s liver; however, it needs further studies to confirm this speculation.

## Conclusion

Our study indicates that [^18^F]AlF-NOTA-PRGD2 PET imaging shows higher tumor-to-background ratio in UM patients. Therefore, [^18^F]AlF-NOTA-PRGD2 tracer has a great advantage in detection of primary and metastatic UM due to its improved sensitivity and specificity compared to [^18^F]FDG tracer; however, this promising pilot data needs more extensive clinical evaluation.

## Data Availability

The datasets generated during and analyzed during the current study are available from the corresponding author on reasonable request.

## Abbreviations

UM: Uveal melanoma
PET: Positron emission tomography
[^18^F]FDG: ^18^F-labelled fluorodeoxyglucose
%ID/g: Percentage of injected dose per gram of tissues
MRI: Magnetic resonance imaging
PET/CT: Positron emission tomography/computerized tomography
HPLC: High performance liquid chromatography
FBS: Fetal bovine serum
P/S: Penicillin/streptomycin
RIPA: Radio immunoprecipitation assay
ROI: Regions of interes
